# The Effect of Polypharmacy on the Charlson Comorbidity Index and Katz Index in Aging People with and without Diabetes Mellitus

**DOI:** 10.5152/eurasianjmed.2021.20070

**Published:** 2021-06

**Authors:** Gulcin Sahingoz Erdal, Hakan Kocoglu, Faruk Karandere, Pinar Kasapoglu, Nilgun Isiksacan, Mehmet Hursitoglu

**Affiliations:** 1 Department of Internal Medicine, Bakırköy Dr Sadi Konuk Training And Research Hospital, İstanbul, Turkey; 2 Department of Biochemistry, Bakırköy Dr Sadi Konuk Training And Research Hospital, İstanbul, Turkey

**Keywords:** Aging, Diabetes mellitus, Index, Polypharmacy

## Abstract

**Objective:**

The prevalence of diabetes mellitus is growing worldwide, as well as in the aging population, and its comorbidity and mortality rates are higher in aging people than they are in young people. It has been observed that the number of drugs used increases in aging patients, especially in diabetic patients. This study aimed to investigate the relationship between polypharmacy and modified Charlson Comorbidity Index (CCI) and Katz Index of Independence in Activities of Daily Living (Katz ADL) scores in aging diabetic and non-diabetic patients.

**Materials and Methods:**

This prospective study included 184 diabetic and 62 non-diabetic subjects who were ≥65 years old. Comorbidity was determined with CCI, and dependency on daily basic activities was assessed with Katz ADL.

**Results:**

CCI and the number of drugs were significantly higher in diabetic groups (*P* = .001). In all subjects and in the diabetic group, there was a negative correlation between CCI and Katz ADL (r = −0.343, *P* = .001; r = −0.383, *P* = .001, respectively); there was a positive correlation between CCI and number of drugs (r = 0.430, *P* = .001; r = 0.248, *P* = .001, respectively).

**Conclusion:**

We found an increase in the number of drugs taken by the aging patients, positively correlated with the CCI score. The increase in the number of drugs used is closely related to the insufficiency in daily life activity and comorbidity, and this predicts 10-year survival. Patients should be directed to special centers or physicians who will be scheduled for multidisciplinary treatment for the prevention of polypharmacy, especially in the aging.

## Introduction

It is estimated that by 2050, the aging population will rise from 900 million to 2 billion people, which means that the proportion of the world’s population over 60 years old will nearly double between 2015 and 2050, from 12% to 22%.^1^ The prevalence of diabetes mellitus (DM) is growing worldwide^2^, as well as in the aging population, and its comorbidity and mortality rates are higher in aging people than they are in young people.^2^,^3^ A recent study conducted in the United States showed that DM affects 10.9 million people aged 65 years and older, and it is expected that this number will reach 26.7 million by 2050, representing 55% of all diabetes cases.^4^

Aging patients are more susceptible to issues caused by an increased number of drugs, which is called polypharmacy, than young people. Yet, there has been a considerable increase in polypharmacy in the geriatric population.^5^ In the United States, the number of prescribed medicines for aging people doubled between 1988 and 2010^6^. Adverse drug reaction–related emergency department visits and hospitalizations, which are commonly caused by polypharmacy^7^, are extremely high in aging people, especially those aged 80 years and older.^8^

In the hope of improving quality of life on newly developed drugs, more and more drugs are given to patients, especially elderly patients. We think that multidrug use may decrease quality of life and negatively affect the survival of aging patients. When we look at the geriatric patient population that we followed in our outpatient clinic, we found that DM was a very comorbid disease because the frequency of diabetes increases with age and we thought that the number of drugs used in these patients may be greater and that diabetic patients may be more exposed to the risk of polypharmacy. In this prospective study, we aimed to investigate the relationship between polypharmacy, the modified Charlson Comorbidity Index (CCI), and the Katz Index of Independence in Activities of Daily Living (Katz ADL) to determine whether polypharmacy affects CCI and/or Katz ADL scores in aging diabetic and non-diabetic patients.

## Materials and Methods

This prospective study was conducted from January 2017 to January 2018 after receiving institutional ethical approval from Ethics Committee of Bakirkoy Dr. Sadi Konuk Training and Research Hospital (2018/336). The study included 184 diabetic (group 1) and 62 non-diabetic (group 2) subjects who were 65 years or older and visited our Diabetes or Internal Medicine outpatient clinics. The non-diabetic patient group consisted of patients who had not previously been diagnosed with DM type 2, had a glycolyzed hemoglobin (HbA1c) level below 6.5%, and had a fasting glucose level below 140 mg/dL. Written consent was obtained from all participants. Before obtaining blood samples, data (including height, weight, age, the number and type of drugs taken by the patients, arterial blood pressure, and diabetes age [in the diabetic group]) of the patients enrolled in the study were recorded on standardized forms.

CCI, a prognostic assessment tool, is based on comorbid conditions with varying assigned weights, resulting in a composite score; it predicts 10-year survival in patients with multiple comorbidities.^9^ Katz ADL is a functional measurement instrument that aims at assessing the status of independence in daily activities.^10^ Patients’ comorbidity was determined with CCI, and dependency concerning basic activities of daily living was assessed with Katz ADL.^10^,^11^ Polypharmacy was defined as the administration of ≥5 drugs for more than 90 days. During the administration of both questionnaires, a nurse who was blinded to the study groups was readily available to assist the patients if required.

Statistical analyses were conducted with SPSS version 23 (IBM SPSS Corp.; Armonk, NY, USA). Mean, standard deviation, median, minimum, and maximum values of variables were given for descriptive calculations between groups. Mann-Whitney U test was used in comparison of two independent groups for variables that did not display normal distribution, and Spearman’s rank correlation coefficient was used to determine the relationships between variables. Student’s *t* test was used for comparing two independent groups in variables with normal distribution. A *P*-value < .05 was considered significant.

## Results

As mentioned previously, group 1 consisted of 184 (129 women) and group 2 consisted of 62 (46 women) subjects with mean ages of 71.15 ± 5.28 years and 71.58 ± 5.35 years, respectively (*P* = .472). There was a significant difference between the two groups regarding HbA1c, random urine albumin/creatinine ratio, high-density lipoprotein cholesterol (HDL-C), low-density lipoprotein cholesterol (LDL-C), dual-energy X-ray absorptiometry, and serum iron, magnesium, hemoglobin, and albumin levels (for *P*-values, see Table 1).

Comparing diabetic patients with non-diabetic subjects regarding the Katz ADL, CCI, and number of drugs that the participants took showed that CCI and the number of drugs were significantly higher in the diabetic group (*P* = .001 for both). Katz ADL did not differ between the groups (*P* = .059) (Table 2).

In all subjects, CCI had a moderate positive correlation with age (n = 246, *r* = 0.525, *P* = .001) and weak positive correlations with HbA1c (n = 246, *r* = 0.322, *P* = .001) and random urine albumin/creatinine ratio (n = 246, *r* = 0.218, *P* = .001). In addition, CCI had weak negative correlations with glomerular filtration rate (GFR) and serum magnesium, iron, and hemoglobin levels. Katz ADL had weak positive correlations with GFR and serum magnesium and iron levels; in addition, it had a weak negative correlation with HDL-C levels. Number of drugs had a weak positive correlation with HbA1c and a weak negative correlation with GFR, LDL-C, and serum magnesium, iron, and hemoglobin levels (see Table 3 for *r* and *P*-values).

In all subjects, there was a negative correlation between CCI and Katz ADL (n = 246, *r* = −0.343, *P* = .001); in addition, there was a positive correlation between CCI and number of drugs (n = 246, *r* = 0.430, *P* = .001; Table 4).

Similarly, in diabetic patients, there was a negative correlation between CCI and Katz ADL (n = 184, *r* = −0.383, *P* = .001); moreover, there was a positive correlation between CCI and number of drugs (n = 184, *r* = 0.248, *P* = .001). In addition, age of DM diagnosis had positive correlations with CCI and number of drugs (n = 184, *r* = 0.248, *P* = .001; Table 4).

## Discussion

In this study, we determined that the number of drugs taken by the patients had a statistically significant positive association with CCI in aging people with and without DM, and this finding allowed us to deduce that polypharmacy could have a negative influence on 10-year survival in aging individuals with different morbidities.

New treatments bring an increase in the number of drugs used in the geriatric population. Polypharmacy causes more adverse reactions in the aging because they are more prone to drug–drug reactions.^11^ In addition, the increasing prevalence of diabetes, along with the aging of the population, draws more attention to polypharmacy and its consequences. For this reason, we wanted to investigate the effect of polypharmacy on comorbidity in aging people by using the prognostic and daily activity assessment tools of CCI and Katz ADL.

According to previous studies, CCI can be a predictor in aging people for perioperative mortality^12^, short- and long-term mortality in hospitalization because of acute illness^13^, short- and long-term mortality in the non-surgical emergency department^14^, survival in acute respiratory distress syndrome^15^, mortality in *Staphylococcus aureus* bacteremia^16^, survival in multiple myeloma^17^, and mortality and functional outcome in ischemic stroke^18^. From the perspective of our results and previous studies’ findings, it can be thought that polypharmacy could have an effect on mortality in the situations mentioned above. We think that further studies are warranted in this field.

A recent study conducted by Lim^19^ assessed the association between Katz ADL and polypharmacy, and it did not show a relationship between them. Another study with a smaller sample size was also unable to determine a significant difference in functional status according to the decrease in the number of medications.^20^ In accordance with the above studies, in our research, we were similarly unable to show a relationship between Katz ADL and the number of drugs taken by the patients in both the diabetic and non-diabetic groups. This result could also have been affected by the patient selection, as outpatient clinics were the source of our study population; it can be assumed that, if these patients can visit the outpatient clinics, they could have higher Katz ADL scores than those who cannot visit outpatient clinics.

Studies have revealed the clinical results of polypharmacy very well in the geriatric population. Multiple drug use has been associated with the development and worsening of geriatric syndromes, such as cognitive impairment, delirium, falls, frailty, incontinence, and weight loss.^21^ Because the presence of geriatric syndromes will correlate with the increase in score in the CCI and Katz ADL scales; these findings support the results we obtained in our study.

Studies have shown that the use of various drugs together by elderly patients can significantly contribute to the occurrence of adverse reactions. It is estimated that the risk of adverse reactions increases by around 50% when 5 drugs are used, and the risk of adverse reactions increases above 95% when 8 or more drugs are used.^22^,^23^ Considering that adverse reactions affect a person’s quality of life and survival, these data support the findings in our study.

The small number of participants is one of the important limitations of this study. Although our study’s design was a prospective one, its data were cross-sectional. Thus, we could not follow up and determine the fate of the study participants (from future morbidity and mortality points of view). Similar studies conducted in the future may focus on these issues.

The increase in the number of drugs taken by aging patients with and without diabetes is positively correlated with CCI score. As we know, this scoring system is being used as a predictor of 10-year survival in patients with multiple comorbidities. Thus, polypharmacy could be a quick determinant of prognosis in such a group of patients. Further detailed studies are needed to verify this.

**Main Points**

- Polypharmacy decreases quality of life and affects the survival of aging patients.
- The number of drugs taken by the patients had a statistically significant positive association with modified Charlson Comorbidity Index in aging people with and without diabetes mellitus.
- Patients should be directed to special centers or physicians who will be scheduled for multidisciplinary treatment for the prevention of polypharmacy, especially in the aging.

## Figures and Tables

**Table 1:** Descriptive Data of Participants and Their Comparisons According to Groups

n = 246	Non-diabetic (n = 62)		Diabetic (n = 184)		*P*-value
	
	Mean ± SD	Med. (Min.–Max.)	Mean ± SD	Med. (Min.–Max.)	
Age, years	71.15 ± 5.28	70 (65–86)	71.58 ± 5.35	70.5 (63–94)	.472
History of diabetes, year	—	—	12.83 ± 8.78	10.5 (1–42)	—
HbA1c, %	5.75 ± 0.32	5.7 (5–6.4)	7.36 ± 1.71	6.8 (5.3–17)	<.001
GFR, mL/min/1.73m^2^	80.13 ± 14.32	84.5 (48–105)	76.59 ± 18.18	82 (21–108)	.321
Random urine ACR, mg/g	13.82 ± 20.29	6.55 (1–129.4)	142.13 ± 575.59	17.35 (0.5–6728)	<.001
TG, mg/dL	148.55 ± 70.15	137.5 (61–501)	164.99 ± 75.76	149 (15–513)	.07
HDL-C, mg/dL	52.92 ± 11.89	51.5 (34–88)	49.52 ± 11.92	48 (27–88)	.047
LDL-C, mg/dL	152.02 ± 32.71	152 (80–231)	135.41 ± 44.15	135 (41–244)	.007^a^
Ca, mg/dL	9.73 ± 0.44	9.7 (7.9–10.6)	9.77 ± 0.55	9.8 (6.1–12.1)	.318
Mg, mg/dL	1.98 ± 0.15	2 (1.6–2.2)	1.83 ± 0.22	1.9 (1.2–2.3)	<.001
Vitamin D, ng/mL	16.87 ± 8.05	16 (6–42)	18.41 ± 14.03	14 (2.4–94)	.682
Fe, μg/dL	81.08 ± 31.26	76 (36–190)	70.95 ± 26.45	66 (18–145)	.037
Hb, g/dL	13.39 ± 1.22	13.35 (10.8–17.2)	12.81 ± 1.53	12.8 (7.6–16.9)	.007^a^
Albumin, g/dL	4.05 ± 0.27	4 (3.1–4.6)	4.18 ± 0.35	4.2 (2.6–5)	.001
BMI, kg/m^2^	28.49 ± 4.14	27.6 (20.7–41.5)	29.36 ± 4.07	29.1 (19–41.3)	.056

*P*-values calculated with Mann-Whitney U test, unless otherwise indicated.

a Student’s *t* test.

ACR, albumin/creatinine ratio; BMI, body mass index; Fe, iron; GFR, glomerular filtration rate; Hb, hemoglobin; HbA1c, glycolyzed hemoglobin; HDL-C, high-density lipoprotein cholesterol; LDL-C, low-density lipoprotein cholesterol; Ca, calcium; Max., maximum; Med., median; Mg, magnesium; Min., minimum; SD, standard deviation; TG, triglyceride.

**Table 2:** Comparisons of Katz ADL, CCI, and Number of Drugs Taken by the Participants Between Groups

n = 246	Non-diabetic (n = 62)		Diabetic (n = 184)		*P*-value
	
	Mean ± SD	Med. (Min.–Max.)	Mean ± SD	Med. (Min.–Max.)	
Katz ADL	5.94 ± 0.4	6 (3–6)	5.83 ± 0.55	6 (2–6)	.059
CCI	2.89 ± 1.03	3 (2–6)	4.5 ± 1.49	4 (2–9)	<.001
NoDs	2.31 ± 1.79	2 (0–8)	5.31 ± 2.58	5 (0–13)	<.001

*P*-values calculated by Mann-Whitney U test.

CCI, Modified Charlson Comorbidity Index; Katz ADL, Katz Index of Independence in Activities of Daily Living; Max., maximum; Med., median; Min., minimum; NoDs, number of drugs; SD, standard deviation.

**Table 3:** Correlation Analysis Between Patients’ Descriptive Data and Katz ADL, CCI, and Number of Drugs Taken by Participants in All Groups

		Katz ADL	CCI	NoDs
Age, years	r	−0.174	0.525	0.106
	p	0.006	<0.001	0.1
HbA1c, %	r	−0.055	0.322	0.26
	p	0.394	<0.001	<0.001
GFR, mL/min/1.73 m^2^	r	0.116	−0.384	−0.166
	p	0.07	<0.001	0.01
Random urine ACR, mg/g	r	−0.011	0.218	0.042
	p	0.863	0.001	0.514
HDL-C, mg/dL	r	−0.206	−0.055	−0.083
	p	0.001	0.392	0.194
LDL-C, mg/dL	r	−0.028	−0.082	−0.152
	p	0.659	0.199	0.018
Mg, mg/dL	r	0.242	−0.248	−0.252
	p	<0.001	<0.001	<0.001
Fe, μg/dL	r	0.169	−0.166	−0.194
	p	0.008	0.009	0.002
Hb, g/dL	r	0.122	−0.227	−0.195
	P	0.057	<0.001	0.002

Values calculated by Spearman’s rank correlation. r range: 0.90–1.00 Very high; 0.70–0.89 High; 0.50–0.69 Medium; 0.30–0.49 Low; 0.00–0.29 weak correlation. If correlation coefficiency is negative (−) that means is negative correlation. A p-value less than 0.05 is statistically significant. Statistically significant values are marked in bold. ACR, albumin/creatinine ratio; CCI, modified Charlson Comorbidity Index; Fe, iron; GFR, glomerular filtration rate; Hb, hemoglobin; HbA1c, glycolyzed hemoglobin; HDL-C, high-density lipoprotein cholesterol; Katz ADL, Katz Index of Independence in Activities of Daily Living; LDL-C, low-density lipoprotein cholesterol; Mg, magnesium; NoDs, number of drugs; p, probability value; r, Spearman’s rank correlation coefficients

**Table 4:** Correlation Analysis Between Katz ADL, CCI, and Number of Drugs Taken by Participants in All Groups and Diabetic Group

		Diabetic				All groups		
	
		Age of DM	Katz ADL	CCI	NoDs	Katz ADL	CCL	NoDs
Age of DM	r	1	−0.106	0.168	0.248	—	—	—
	p	—	0.15	0.023	0.001	—	—	—
Katz ADL	r	−0.106	1	−0.383	−0.056	1	−0.343	−0.072
	p	0.15	—	<0.001	0.453	—	<0.001	0.262
CCI	r	0.168	−0.383	1	0.248	−0.343	1	0.430
	p	0.023	<0.001	—	0.001	<0.001	—	<0.001
NoDs	r	0.248	−0.056	0.248	1	−0.072	0.430	1
	p	0.001	0.453	0.001	—	0.262	<0.001	—

Values calculated by Spearman’s rank correlation. r range: 0.90–1.00 Very high; 0.70–0.89 High; 0.50–0.69 Medium; 0.30–0.49 Low; 0.00–0.29 weak correlation. If correlation coefficiency is negative (−) that means is negative correlation. A p-value less than 0.05 is statistically significant. Statistically significant values are marked in bold. CCI, modified Charlson Comorbidity Index; Katz ADL, Katz Index of Independence in Activities of Daily Living; NoDs, number of drugs; p, probability value; r, Spearman’s rank correlation coefficients.
